# Behavioural and EEG correlates of forward and backward priming—An exploratory study

**DOI:** 10.1371/journal.pone.0322930

**Published:** 2025-05-08

**Authors:** Mareike Wilson, Marc Wittmann, Jürgen Kornmeier

**Affiliations:** 1 Institute for Frontier Areas of Psychology and Mental Health (IGPP), Freiburg, Germany; 2 Department of Psychiatry and Psychotherapy, Medical Center, University of Freiburg, Freiburg, Germany; 3 Faculty of Medicine, University of Freiburg, Freiburg, Germany; Sapienza University of Rome: Universita degli Studi di Roma La Sapienza, ITALY

## Abstract

During affective priming, perception of an emotional “prime stimulus” influences the reaction time to the subsequent emotional “target stimulus”. If prime and target have the same valence (congruent trials), reactions to the target are faster than if prime and target have different valences (incongruent trials). Bem introduced a backward priming paradigm in 2011, where first the target was presented and then the prime after the response. Similar to the classical affective forward priming effects, he found faster reaction times in congruent compared to incongruent trials, and interpreted these results as evidence supporting precognition. In the present exploratory study, while measuring EEG, we combined a forward priming paradigm with a related backward priming paradigm, following Bem’s study. We analysed the EEG data on a group level (ERPs) and on an individual level (single participants, applying artificial neural networks). We found significantly faster reaction times for congruent compared to incongruent trials in the forward priming experiment (p = 0.0004) but no statistically significant differences in the backward priming experiment (p = 0.12). We also found significant differences in ERP amplitude in the forward priming congruent vs incongruent conditions (P8 electrode: p = 0.0002). Backward priming results show weaker, shorter, and less significant differences between congruent and incongruent trials, with maxima at electrodes P7, P3, CP5, and CP1. The neural network results were very variable across participants in both the backward and forward priming and on average, the accuracy results were at chance level for both the forward priming as well as the backward priming. Our results replicate behavioural findings and extend the EEG findings for forward priming. We did not replicate Bem’s backward priming results. These exploratory EEG results are weak, however they give a good starting point for future studies.

## Introduction

Priming is a phenomenon where perception of a “prime stimulus” influences the processing of the subsequent “target stimulus” and participants’ reaction times. During affective priming, the prime and the target have emotional content with positive or negative valence [1]. In a typical affective priming task, participants judge as quickly as possible whether an image—the target—is pleasant or unpleasant, by pressing one of two assigned buttons on the keyboard. Before the image is presented, a positive or negative word (e.g., “beautiful” or “ugly”)—the prime—is shown briefly on the screen. On average, participants respond with faster reaction times when the valence (positive or negative) of the prime word and the target image are congruent than when they are incongruent [2]. This classic affective priming effect is interpreted as working in a highly automated fashion [2,3]. In the following we call this the “forward priming effect”.

Some studies recorded participants’ electroencephalogram (EEG) in order to investigate the neural processes underlying various types of forward priming using different stimuli [4–7]. One approach to analyse EEG data is the calculation of ERP (event-related potential) traces, which result from averaging across many trial repetitions. Forward priming effects were reported for two ERP components. The first ERP component is a negative deflection around 400 ms after target onset and located at centro-parietal electrodes (“N400”). The second component is a centro-parietally distributed “Late Positive Potential” between 400–700 ms after target onset. Both components show larger amplitudes in semantically incongruent compared to congruent trials [4–7].

In “backward priming” (also known as retroactive priming), the typical sequence of prime and target is reversed. First, a positive or negative target image is presented and the participant presses one of the two assigned buttons for the respective valence. After a button press, a prime word with negative or positive valence occurs on the screen. In the seminal study by Bem [8], the typical reaction time effects, as found with forward priming, also occurred with backward priming. That is, when the target image and the prime word were congruent, the recorded reaction times were shorter than in the incongruent case. Since the prime appears after the button press, it cannot affect reaction time in an obviously causal way. In contrast, the future word (the prime) seems to retroactively affect reaction to the target image in the present, or so Bem’s interpretation. These results are labelled as precognition effects pertaining to a time reversal of the typical order of events. Precognition is the proposed ability to perceive or sense events or, more general, information at the present moment, although this information will only be generated in the future [9,10]. A number of studies have been done investigating precognition. Some of the studies find significant precognition effects like Bem did [11–13]. A meta-analysis across 15 studies indicated overall significant priming effects when participants partook in backward priming experiments [14]. This is supplemented by a large body of case reports about precognitive experiences [15–18]. However, a persistent debate regarding the validity of the supporting findings prevails since other studies did not find positive results in a variety of precognition or similar anticipation tasks [19–25]. The main methodological criticisms towards such positive findings in the literature, and especially to the publication by Bem (2011), pertain to (1) too liberal statistical analyses methods, (2) selective data collection (the file drawer problem), and (3) general bad scientific practices associated with the studies [26,27].

In the present study, we combined a classical affective forward priming paradigm with a related backward priming paradigm, following the procedures used by Bem. We additionally measured the EEG while our participants executed the forward and backward priming tasks. Our main interest was to test the following research questions:

1. Can we replicate the behavioural and EEG results from the forward priming literature using the present prime-word, target-image paradigm?
2. Can we replicate the backward priming results from the Bem study?
3. Can we find EEG signatures related to backward priming, and if yes, are they comparable to the forward priming EEG effects?

For the EEG analysis, we used an exploratory, hypothesis-generating approach including artificial neural network methods, as will be explained and motivated below.

Personality traits can be defined as a consistent pattern of an individual’s behaviour which influences current experience, including exceptional experience and behaviour [28]. An individual’s ability to remain focused on mental experiences and avoid distractions from the task will in general be related to any performance but also related to psi experience. Regarding trait-related influences on forward and backward priming, we assessed mindfulness, impulsivity, and the presence of exceptional experiences in one’s life. Specifically, mindfulness and impulsivity are two end points on a two-dimensional scale of self-regulation and are opposite in their influences on cognitive and emotional processing, i.e., more and less focused and sustained attention, respectively [29]. Since mindfulness and impulsivity as individual traits can have a positive and negative effect on attentional processing, respectively, and thus on performance, we chose to assess these dimensions of personality. The presence of exceptional experiences might be an indicator of how people perform in a precognitive task, as has been shown before [30].

For both forward priming and backward priming, we hypothesize that participants will have faster reaction times for the congruent trials compared to the incongruent trials.

## Materials and methods

### Participants

Forty-three participants with an age range between 18 and 36 years (mean age: 23.1 years, S.D.: 3.4; 13 males, 30 women) who spoke fluent German took part in the priming study. Due to the fact that participants were recruited through an online portal system for advertising mini-jobs for students and through paper notices at the University of Freiburg, the majority of them were university students. The recruitment of participants for this study started on the 6^th^ of May 2022 and ended on the 22^nd^ of December 2022. Participants received a financial compensation of €10 per hour. To avoid a cognitive bias in the participants, the parapsychological nature of the retroactive task was only revealed to them once the experiment was over. The study was approved by the local ethics committee of the Institute for Frontier Areas of Psychology and Mental Health (IGPP_2022_15). Before inviting participants to the experiment, we conducted an interview on inclusion and exclusion criteria in order to decide whether the participants were eligible to take part in the experiment. This included questions about whether they were in good health and had no known neurological or psychiatric problems. The participants provided written informed consent prior to starting the experiment. Due to the mistakes in the task and/or technical reasons, twelve participants were removed from the analysis resulting in a total of 31 participants.

### Questionnaires

The PExE-II assesses the presence and frequency of exceptional experiences in the participant’s life. The questionnaire part of interest consists of 20 items and classifies these experiences in four factors (external phenomena, internal phenomena, phenomena of coincidence, and of psychophysical dissociation), each of which is assessed by 5 items scoring on 5-level Likert scales. 2) The Barratt Impulsiveness Scale (BIS-11, [31]) consists of 30 4-point items ranging from 1 (rarely) to 4 (almost always). The employed German version was validated by Preuss et al. (2008, [32]) and it is recommended to use the overall sum score. 3) The Freiburg Mindfulness Inventory (FMI, [33]) measures mindfulness on the basis of a two-dimensional structure with the factor ‘presence’ referring to the ability to attend to the present moment (“I am open to the present moment”) and the factor ‘acceptance’ referring to a non-judgmental attitude (“I am able to smile when I notice how I sometimes make life difficult”). A 14-item version has been developed which was used here.

### Experimental paradigm

Participants took part in two experiments in total. Experiment 1 contained a forward priming task, Experiment 2 a backward priming task. Each experiment contained 400 trials that were split up into 12 blocks. This resulted in 11 blocks of 36 trials and 1 block of 4 trials. In both experiments the priming task contained a priming stimulus and a target stimulus. The priming stimulus was a positive or a negative word and had to be semantically relevant to the target stimulus. In congruent trials prime and target had the same valence, in incongruent trials they had different valences. The participants were instructed to read this word. The target stimulus was a picture with either positive or negative valence. Participants had to observe the picture and indicate by pressing one of two possible (left, right) keys whether the observed picture was of positive or negative valence. Due to the fact that we needed 400 images, the pictures were taken from 3 image datasets: the International Affective Picture System (IAPS) [34], the Open Affective Standardized Image Set (OASIS) [35], and the Racially Diverse Affective Expression (RADIATE) face stimulus set [36]. For the OASIS dataset, we only used images that were within a certain range of valence values (each image in the OASIS database is associated with a valence value). We removed the extreme images (e.g., those of bloody wounds, mutilated or dead people, etc.) and neutral images in the following way: the valence range was determined by calculating the mean valence of all the images in the dataset and taking the mean ± 1.5 as the lower and upper thresholds. Through manual selection the very extreme negative images were removed. Lastly, for the RADIATE dataset, only the happy and fearful images were used. All images used in the forward priming experiment were also used in the backward priming experiment to allow for comparison. 50% of the images were positive and 50% were negative in valence. The prime words were chosen based on Wittmann et al.’s [37] German translation of the English words used by Bem [8]. Whether or not a prime was negative or positive in valence was determined by a random number generator [38] where there was a 50% chance that a prime would be negative or positive. This resulted that, on average, 50% of all trials were congruent and 50% incongruent. The experimental paradigm was coded using the PsychoPy package (version 2023.1.2) [39] and Python 3.8.10.

### Forward priming experiment

**Fig 1:**
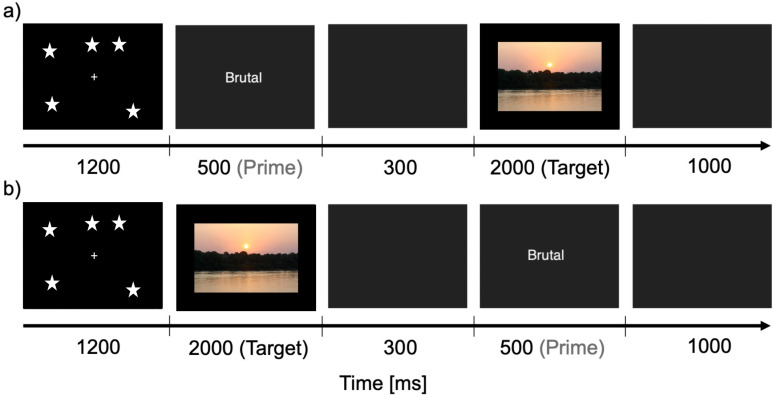
Experimental paradigm and stimuli in (a): Forward priming and (b): Backward priming. (a): The image of galaxies (for demonstration purposes a schematic is depicted here) appeared for 1200 ms, after which the prime word was shown for 500 ms. The prime word was followed by an inter-stimulus interval (ISI) of 300 ms, where a blank was presented. After the ISI the target image (for demonstration purposes a private image is depicted here) was presented and the participant had to respond within the time duration of up to 2000 ms whether the target was of positive or negative valence. As soon a participant would press a button, the target stimulus was replaced by a blank screen, which was presented for 1000 ms and ended the trial. Backward priming trials (b) sequences were the same, with the exception of replacing the positions of target and prime with each other.

As shown in Fig 1a, in one trial of the forward priming experiment, participants observed the Hubble image “Hubble Ultra Deep Field” [40] on the screen for 1200 ms marking the onset of the trial, then 500 ms of a prime word, followed by an interstimulus interval (ISI) of 300 ms, and then a target image was presented. After participants had pressed one of the two response keys or alternatively after 2000 ms, a black screen was shown for 1000 ms. This forward priming experiment thus differed between congruent and incongruent trials concerning valence of the prime word and target image. In a congruent trial, the prime word and target picture had the same valence (positive prime + positive target/negative prime + negative target). In an incongruent trial, the prime word and the target picture had different valences (positive prime + negative target/negative prime + positive target).

### Backward priming experiment

The backward priming experiment had the same structure as the forward priming experiment with one major exception. Here, the order of prime and target stimulus was changed. The target picture followed directly after the Hubble image [40]. After participants had pressed one of the two response keys or after maximally 2000 ms, the blank screen ISI occurred for 300 ms followed by the prime word, which was presented for 500 ms (Fig 1b). Correspondingly, the backward priming experiment also differed between congruent and incongruent trials. Here, a congruent trial consisted of a positive target + positive prime or a negative target + negative prime. An incongruent trial consisted of a positive target + negative prime or a negative target + positive prime.

### EEG recording

The electroencephalogram (EEG) was recorded using 32 active silver/silver chloride electrodes using with the extended 10–20 system of electrode positions [41]. This was done using the BrainVision ActiCHamp amplifier. The data was digitized at a sampling rate of 1000 Hz and online bandpass filtered at 0.01–120 Hz. The impedances were kept below 10 kΩ for all electrodes during the experiment.

### EEG pre-processing

All pre-processing was done using the MNE-Python package (1.2.1) [42] in Python 3.9.6. The raw data was offline band-pass filtered from 0.1 to 30 Hz and re-referenced to the common average across all electrodes. Due to technical reasons, we did not have vertical electrooculogram (vEOG) electrodes to determine eye blinks. Therefore, we used the Fp1 electrode for eye blink detection. The default settings from the MNE package were used for the pre-processing of this electrode for the eye blink detection.

As is standard in the MNE package, an Independent Component Analysis (ICA) was done to detect the eye blinks. The Independent Components (ICs) were correlated with the Fp1 electrode. If a component correlated with the electrode more than *r *= 0.5 it was labelled as an IC related to eye movements and was removed from the data. This threshold was determined after manual inspection of the IC correlations. Across participants, the average number of components labelled as eye blinks and thus removed was 1 (standard deviation = 2.4). For the remaining artifacts, an artifact rejection threshold of ± 100 μV (peak-to-peak amplitude) was defined. The data was epoched to only incorporate the time window during which the target was presented for both the forward as well as the backward priming experiment. Data was baseline corrected, with the average amplitude in a time window between 60 ms before and 40 ms after stimulus onset of the target [43].

### Procedure

An introduction and the opening instructions given to participants upon entering the laboratory informed them about the testing of the priming effect. Subsequently, the participant read and signed the consent form and filled out the trait questionnaires. After the participants finished filling out the questionnaires, the experimenter instructed the participants about the task and ensured that the instructions given were similar across participants. The experiment took place in an EEG cabin. An example trial of the forward priming and the backward priming was shown to give the participant an idea of the sequence order of the stimuli. Then the experiment started. Participants were given the choice to have a break after every block. After the 12^th^ block and the end of the first experiment, the experimenter entered the EEG cabin and checked on the impedances of the electrodes. During this time the participant had a predefined break. Then Experiment 2 started and participants were again given the choice of having breaks in between blocks. For half of the participants the forward priming experiment started first, followed by the backward priming experiment. The other half of the participants started with the backward priming experiment. Additionally, the buttons associated with negative and positive were also counterbalanced across participants. The buttons associated with negative and positive were kept the same within one participant’s measurement.

### Data analysis

The reaction times were calculated by separating congruent and incongruent trials. Trials were labelled congruent or incongruent depending on the valence of the target image and the valence of the randomly chosen prime. Only trials where participants correctly classified the valence of the image (according to the validation of the respective image datasets [34,35]) within the given time range were used for the analysis. This resulted in some participants being excluded from the analysis, as they incorrectly classified images. The time range for a valid reaction time was 250 ms after target onset up to 2000 ms. For more information on the trials that were incorrectly classified, see S1 Table.

#### Reaction times.

The reaction times of the congruent and incongruent trials were calculated for every trial and every participant as the difference between the time the correct button was pressed and onset of the target stimulus. Next, the median reaction time of the trials was calculated for every participant. To get contrasts between congruent and incongruent conditions, the respective median reaction times per subject were subtracted from each other (congruent minus incongruent). To test whether or not there are statistically significant differences a t-test was conducted. Given the a priori skewness of reaction time data, we additionally calculated a nonparametric Wilcoxon test. Lastly, we also calculated a Bayes Factor using the Pingouin package [44]. Correcting for multiple testing was done using the Bonferroni-Holm method [45].

#### Event Related Potentials (ERPs).

The ERP analysis was focused on the time window of the target stimulus presentation. Despite the differing durations of the target presentations across trials (the target duration ends as soon as the participant presses the button), we selected a trial time window starting at onset of the target stimulus and lasting 1000 ms. The EEG data were averaged across trials for single participants to result in ERPs and then across participants to result in grand mean ERPs. To get different traces, the grand mean ERP from the incongruent trials was subtracted from the congruent grand mean ERP.

In our exploratory analysis, we calculated paired running t-tests for each single electrode, testing for differences between congruent and incongruent trials and calculating the p-value at every time point. This approach was used to determine spatial and temporal regions of interest. Due to the exploratory nature of our analysis, we did not correct for multiple testing.

The reason for this approach is that we did not have theoretical predictions that would narrow down the high-dimensional data space to a narrow spatial (electrodes) and temporal (time) window for the EEG analysis (i.e., a spatio-temporal regions of interest, “ROI”). The running t-test thus served to find potentially interesting locations and time windows (hypothesis generation) rather than proving that the indicated effects are reliable (hypothesis testing).

#### Neural networks.

The aim of the neural network analysis was to give the network EEG data and see how well the network can label a single EEG trial as congruent or incongruent. The unique characteristic of this analysis is that one does not need to define spatio-temporal regions of interest, as done in classical analyses. For each participant, a network was created and trained to differ between congruent and incongruent trials using the single trial EEG data as defined above. This was done separately for the forward priming and the backward priming, resulting in two networks per person. The data used for the analysis was all the congruent and incongruent single trials (not the difference traces), all the electrodes and a time window of 400–700 ms after stimulus onset. This time window was chosen based on results from previous forward priming EEG studies [4–6]. A 4-fold cross-validation was done with the data. The total EEG trials were separated into four sections, each containing 25% of the trials. In four separate steps, each of the four 75% sections was once used for training the network and the remaining 25% for evaluation of the network. This resulted in four accuracy values that were averaged to a median accuracy value for each participant. This procedure was run three times using three different random seeds (for the initial state of the network) which resulted in three average accuracies per participant. Therefore, each participant had a total of twelve accuracies that were averaged. To ensure the network was trained and evaluated equally on congruent and incongruent trials, the same number of congruent and incongruent trials were used. This means that for the training and testing of every fold 50% of the trials were congruent and 50% were incongruent. These trials were also shuffled for the training.

**Fig 2:**
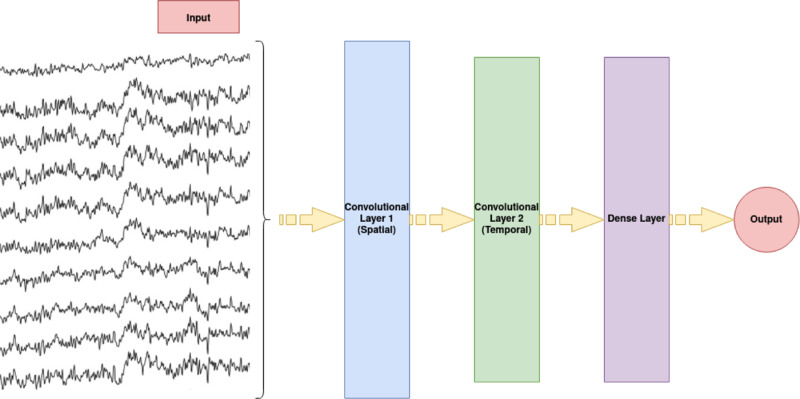
Neural network architecture. Visual depiction of the neural network architecture. The input were single trial EEG traces that included all electrodes and time window of 400 ms to 700 ms after target image onset. The first layer was a convolutional layer with a kernel size matching the number of channels, filtering the data spatially. The second layer was designed to filter the data temporally. The last layer was a dense layer with a sigmoid activation function, resulting in binary classification of the trials (the class of each trial being either congruent or incongruent).

Coding of the network was done using the keras package (version 2.13.1) [46]. A random search encompassing different architectures and hyperparameters was done to obtain the best activation function (relu, softmax, softplus, tanh, selu, and elu), number of filters (four, five, six, seven, and eight), optimizers (adam, sgd, and rmsprop), learning rates (1e-2, 1e-3, and 1e-4) and loss functions (binary crossentropy, poisson, and kl divergence) for the convolutional layers. In total, ten different sets of hyperparameters and architectures were tested before choosing the architecture and hyperparameters that resulted in the best accuracy for every participant. After each layer there was a dropout with a rate of 0.5. The number of epochs was set to 80 with a batch size of 32. The neural network used had a simple architecture of two 2D convolutional layers and one output layer (see Fig 2 for a visual representation of the network architecture). The first layer had a kernel size corresponding to the number of channels. The convolutional layer was designed to spatially filter the data. This reduced the number of channels to the optimum number of filters found during the random search. The second layer had a kernel size of 25 and was designed to temporally filter the data. The last layer was a dense layer with a sigmoid activation to allow for simple binary classification.

## Results

### Reaction times

**Fig 3:**
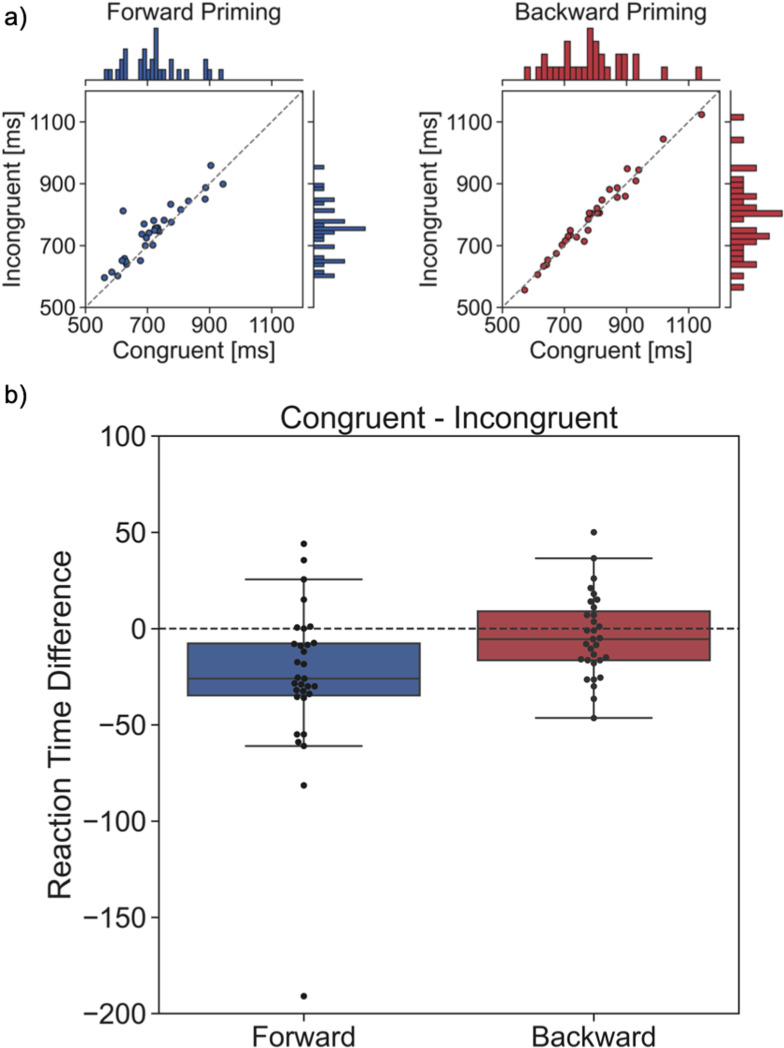
Reaction time results. **(a)** Median reaction times for every participant in milliseconds for the forward priming (in blue) and backward priming (in red). The x-axes depict the congruent trial reaction times and the y-axes the incongruent trial reaction times. The majority of data points in the forward priming condition are above the diagonal indicating overall shorter reaction times for congruent compared to incongruent trials. For backward priming, the data points are about equally distributed among the diagonal line, indicating no effect. **(b)** Box plots with difference reaction times (congruent-incongruent) for the forward and backward priming experiments. Red and blue rectangles indicate median reaction times (central horizontal line) across participants together with upper and lower quartiles. Black circles indicate median reaction times from individual participants. The forward priming box plot shows a clear negative shift, while the backward priming boxplot is close to the zero line.

Fig 3 depicts the reaction time results from the congruent and incongruent conditions of both the forward priming and backward priming experiments. Overall, in the forward priming experiment, participants show significantly shorter reaction times in the congruent trials (median: 720 ms) compared to the incongruent trials (median: 751 ms) (Wilcoxon: statistic = 68.0, p = 0.0004. T-test: statistic = -3.48, p = 0.0008. Cohen’s d = 0.279. BF_10_ = 44). No such systematic difference between conditions was found for the backward priming experiment, as the reaction times in the congruent trials (median: 780 ms) were close to those in the incongruent trials (median: 804 ms) (Wilcoxon: statistic = 188.5, p = 0.12. T-test: t = -0.98, p = 0.17. Cohen’s d = 0.03. BF_10_ = 0.6). Two interesting qualitative observations are the two outliers and an overall larger variability in the backward priming compared to the forward priming experiment. This is even more remarkable because of the within-design of the study (all participants performed both the forward and backward priming task).

### Event Related Potentials (ERPs)

**Fig 4:**
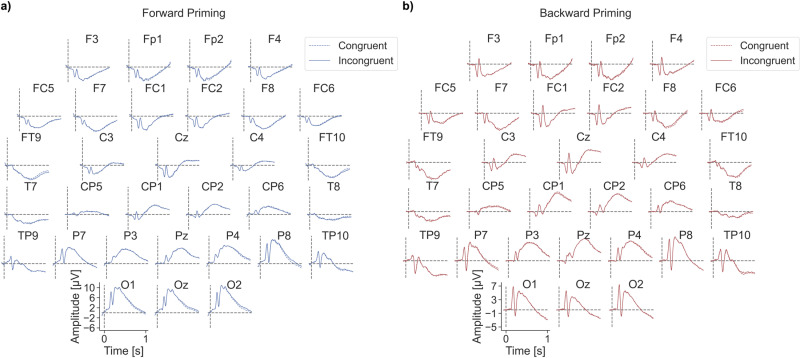
ERP traces. ERP Grand Mean (ERP) traces (congruent = dashed line; incongruent = filled-out line) for every EEG electrode. The forward priming data **(a)** are depicted in blue and the backward priming data **(b)** in red. Each subplot represents one EEG electrode. The x-axis of every subplot shows the time in milliseconds, with zero indicating target stimulus onset. The y-axis shows the amplitude in μV.

**Fig 5:**
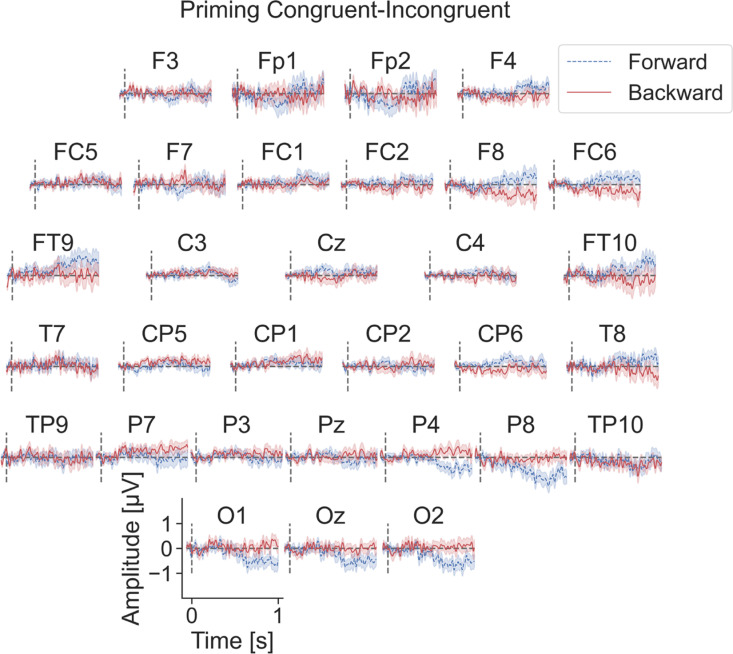
Difference ERP traces. Difference ERP Grand Mean (dERP) traces (congruent – incongruent ± SEM) for every EEG electrode. The forward priming data are depicted in blue and the backward priming data in red. Each subplot represents one EEG electrode. The x-axis of every subplot shows the time in milliseconds, with zero indicating target stimulus onset. The y-axis shows the amplitude in μV.

Fig 4 shows the ERP traces for congruent and incongruent separately. For ease of comparison between forward and backward priming, Fig 5 shows the difference ERPs (dERPs) of congruent minus incongruent ERPs for all electrodes ± standard errors. Blue/red traces correspond to the forward/backward priming experiment, respectively. For most electrodes, the difference between congruent and incongruent is very small. However, for the forward priming traces (blue) there are some obvious deviations from the zero line at the right-hemispheric parietal and occipital electrodes and bilaterally at frontotemporal electrodes. The backward priming results show fewer and less obvious deviations from the zero line at the electrodes P7, P3, CP5, and CP1.

**Fig 6:**
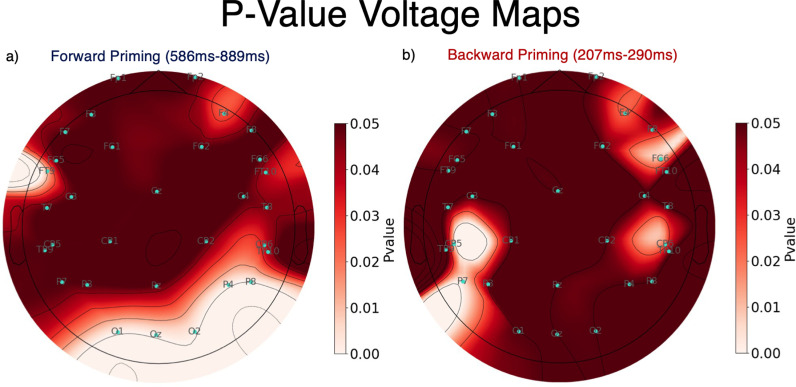
Statistical topographic maps. Depicted are topographic maps showing median p-values for specific time windows both for the forward and backward priming experiments. The right subplot, **(b)**, shows the backward priming p-values 207 ms to 290 ms after stimulus onset of the target image. The left subplot, **(a)**, shows the forward priming p-values from 586 ms until 889 ms after stimulus onset of the target image. The darker the red, the less significant the electrode and the lighter the red, the more significant.

Fig 6 shows topographic maps of p-values for specific time ranges, defined as follows: For every electrode, a running t-test (i.e., a paired t-test for every time point) was calculated, comparing congruent to incongruent trials. A time window was determined where the p-value was below 0.05 for the longest period of time across all electrodes. For the forward priming experiment this was at the P8 electrode and for the backward priming experiment this was at the CP5 electrode. Next, we calculated the minimum p-values in the specific time window where the p-value was below 0.05 for the longest time. For the forward priming, a time window between 586 ms and 889 ms after target image onset was determined and for the backward priming task, a time window between 207 ms and 290 ms after target onset. After the minimum p-value in this time window was found, the p-values 10 ms before to 10 ms after this value were averaged resulting in a median p-value for a 20 ms long range. The topographic maps in Fig 6 represent the spatial interpolations of these median p-value. Additionally, for visualization purposes, we capped the p-values at 0.05, meaning that every p-value larger than 0.05 is represented as 0.05. From these topographic maps it becomes obvious that there are more electrodes that are statistically significant in the forward priming, compared to the backward priming task. In addition to this, most of the significant electrodes are right hemispheric in the forward priming and left hemispheric in the backward priming.

**Fig 7:**
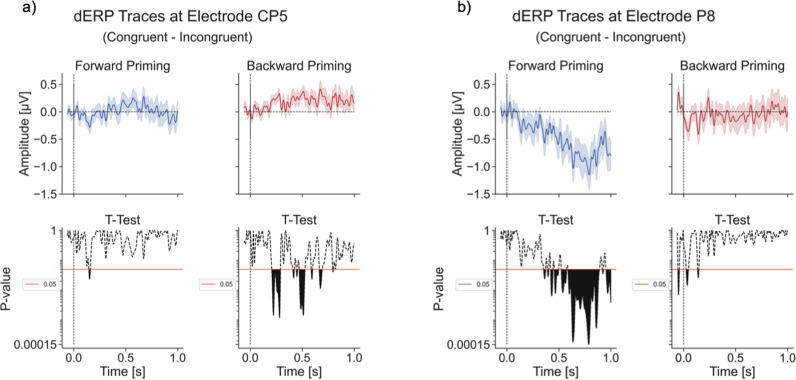
Single electrode difference ERP traces. Difference ERP traces (congruent minus incongruent) at specific electrodes at CP5 **(a)** and at P8 **(b)**. The top graphs in **(a)** and **(b)** show the ERP difference traces (forward priming in blue and backward priming in red) and the bottom graphs show the uncorrected paired t-test results for every time point. The x-axes of all plots are time with zero indicating target stimulus onset (in seconds). The y-axes of the differences traces (top plots of the subplots) show the amplitude in µV while the y-axis of the t-test results (bottom plots of the subplots) shows the p-value logarithmically scaled. The black filled area of the t-test plot shows all p-values smaller than a threshold of α=0.05. Important to note is that the range of the y-axis for the t-test plots is different from **(a)** and **(b)**.

Fig 7 shows the difference traces of the two electrodes with the highest significance in the backward priming (CP5, Fig 7a, S1 Fig) and forward priming experiment, respectively (P8, Fig 7b, S2 Fig). Fig 7a shows that this statistically significant difference is only present for short time windows. The minimum p-value for the backward priming at the CP5 electrode is 0.0012 (Cohen’s d = 0.23), while the amplitudes for the congruent = 0.91 µ V (± 0.25 SEM) and incongruent = 0.59 µ V (± 0.26 SEM). Fig 7b (P8) shows that this statistical significance in the forward priming experiment is present for approximately 400 ms and stronger (smaller p-values) compared to the backward priming results, where the longest period of statistical significance is around 83 ms. The minimum p-value = 0.00015 (Cohen’s d = 0.33) with the congruent amplitude at this timepoint being 0.18 µ V (± 0.63 SEM) and the incongruent amplitude being 1.32 µ V (± 0.61 SEM). In addition to this, the electrodes with most significance differ between forward priming and backward priming experiments.

**Fig 8:**
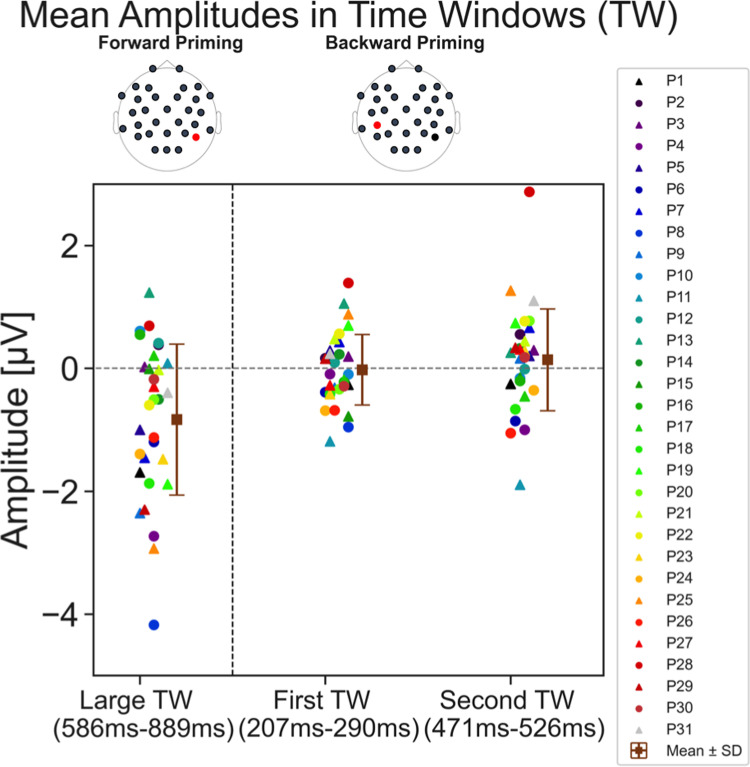
Averaged dERP amplitudes of individual participants. dERP amplitudes averaged across certain time windows. Participant average amplitudes for the P8 forward priming difference trace (left) and CP5 backward priming difference trace (right) for specific time windows. Each participant is depicted in a different colour. The mean amplitude across participants is depicted by the brown square (± standard deviation). The y-axes show the single participant dERP amplitude in µ V. The P8 forward priming time window is the entire length the signal is significantly different (see Fig 7B). At the CP5 electrode, there are two time windows (TW) of the backward priming depicted. The first being the first time window where the p-value is below 0.05 at the CP5 electrode ranging from 207 to 290 ms and the second one being the second, later time window (471 to 526 ms) (see Fig 7A). For all time windows the mean amplitude was calculated and depicted here.

Fig 8 shows the averaged amplitude results from Fig 7 on the level of individual participants for specific time windows. The left side shows the average amplitudes for the forward priming dERP trace at the P8 electrode. Here the time window is 586 ms to 889 ms after target onset (based on the results depicted in Fig 7b). Here we see that most participants show a negative difference between congruent and incongruent. There is also quite a larger variability across participants, as well as two outliers. To the right is the average amplitude of specific time windows with maximal significance, based on the backward priming dERP of Fig 7a. The two time windows chosen were the first time window of significance (207 ms to 290 ms) and the second time window of significance (471 ms to 526 ms). It is clear that around half of the participants show a positive difference between congruent and incongruent amplitudes and the other half a negative difference for both time windows. Here, in the backward priming, there is less variability than compared to the forward priming amplitudes, with the mean difference amplitude being around 0 µ V for both time windows. Nonetheless, there are still outliers (especially in the larger time window of the two). However, doing the analysis without these outliers does not change the overall pattern of the results.

### Neural networks

**Fig 9:**
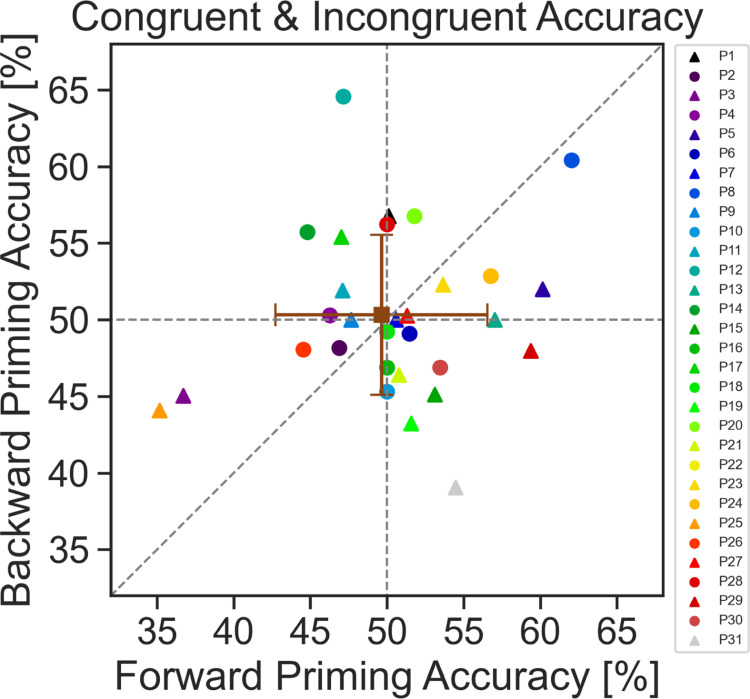
Neural network accuracies. Classification accuracies (congruent versus incongruent trials) of the artificial networks for forward (x-axis) and backward priming (y-axis) for every participant. Each icon represents the result from one participant. The brown square is the grand mean classification accuracy across all participants ± standard deviation. An accuracy value of 50% means that the network performed at chance level. An accuracy below 50% means that the network misclassified more trials than correctly classified them.

Fig 9 shows the results of the neural network trained to classify congruent vs incongruent trials. Every participant had their own neural network trained 12 times (4 folds and 3 different random seeds) resulting in 12 different accuracies which were averaged. The icons in Fig 9 depict the average of those accuracies for each participant. It is clear that across participants there is a large variation in accuracies in both the forward priming as well as the backward priming. The range of accuracies seems to be slightly larger in the forward priming compared to the backward priming. The average accuracy across participants (depicted in brown) is around chance level (50%) for both forward priming and backward priming.

### Questionnaires and correlations with reaction time and ERPs

The results of the PExE, BIS-11, and FMI were correlated with the reaction time difference (Fig 3b) as well as with the single participant dERP results (Fig 8). This was done separately for forward priming and for backward priming. Correlations were done calculating the Pearson as well as the Spearman correlations. For most correlation calculations of the three questionnaires and reaction times there were non-significant correlations. However, for the FMI correlations with the reaction time difference for backward priming, as well as the CP5 first time window amplitudes in backward priming, there were significant results with a Pearson correlation. There was a negative correlation between the Presence questions and the backward priming reaction time difference between congruent and incongruent trials (r = -0.4, p = 0.02) and the FMI Sum and the first time window backward priming amplitudes in the EEG (r = -0.37, p = 0.04). When correlating using the Spearman correlation, these results ended up being non-significant. Results can be seen in the appendix (S2 Table, S3 Fig).

## Discussion

In the present study, we compared reaction time results and EEG recordings from congruent and incongruent conditions in a classical affective forward priming experiment and in a related backward priming experiment. We replicated previous findings of faster reaction times in congruent compared to incongruent forward priming conditions. The reaction times in the backward priming experiment were in the same range as in the forward priming experiment. However, no significant reaction time difference between congruent and incongruent conditions could be observed in the backward priming condition.

To the best of our knowledge, this is the first study that investigated word primes and image targets using EEG. In our forward priming ERP results, we found smaller amplitudes in congruent compared to incongruent trials at parietal electrodes, with a maximal difference at about 600 ms after target stimulus onset. The backward priming experiment revealed two short time windows with significant deviations between congruent and incongruent backward priming conditions. However, due to the exploratory nature of our EEG analysis, these results were not corrected for multiple testing and therefore need to be supported with future studies. Our additional application of neural networks to analyse the EEG data of single participants did not reveal further significant differences.

There were no reliable correlations between the personality questionnaires (frequency of life time exceptional experiences, mindfulness, impulsivity) and the behavioural outcomes. Of note are small correlations in the backward priming experiment using parametric statistics (Pearson correlations) but which we did not replicate using non-parametric calculations (Spearman). The more mindful the participants were, according to Pearson’s correlation coefficients, the smaller the difference between congruent and incongruent trials. If one wanted to interpret these tentative findings, one would conclude that less mindful individuals show a weaker anomalous effect.

### Behavioural forward priming results

We found statistically significant faster reaction times for congruent compared to incongruent trials for the forward priming task. These results fit with previous findings [6,47]. On average, participants’ reaction time difference between congruent and incongruent was 26 ms (with participants reacting faster to congruent trials). The mechanisms underlying this faster reaction time for congruent vs incongruent is hypothesized to be a pre-activation of neural networks representing the affective category (in our case positive or negative) by the prime [48].

### Behavioural backward priming results

Reaction times in the backward priming experiment were in the same range as in the forward priming experiment. However, we found no significantly faster reaction times in the congruent compared to the incongruent backward priming conditions. The data showed a trend towards faster reaction times in congruent vs. incongruent trials (median 5.5 ms difference), similar to the forward priming effects. In Bem’s original study [8], forward priming effects (reaction time difference between trial conditions) were also larger than backward priming effects. One reason for our failure to replicate previous affective backward priming results may be the relatively small number of participants we have here in our exploratory study. This sample size was sufficient to detect forward priming effects, but studies having shown behavioural backward priming effects in the laboratory typically used n = 100 participants due to the smaller effect size [8,37]. Dozens, if not hundreds, of studies have been conducted with experimental paradigms pertaining to the forward priming effect and which are based on a confirmed theory in psychology and cognitive neuroscience. In contrast, the backward priming task has so far been much less studied and is lacking an accepted theory, which makes the interpretation of both positive and negative findings difficult.

### General and specific challenges of EEG data analysis

The analysis of EEG data is a principal challenge because of its high dimensionality (number of channels x number of data points, etc.), thus leading to the statistical problem of multiple testing. Predefining spatio-temporal regions of interest (ROIs) reduces the dimensionality problem and therewith the multiple testing problem. However, ROIs necessitate precise hypotheses about relevant brain areas and time windows of interest. Moreover, restricting the analysis focus on predefined spatio-temporal ROIs prevents the detection of potentially interesting but yet unknown effects outside the ROIs. Additionally, the stimulus set of the current study contains a highly diverse set of pictures, resulting in different types of processing. Due to the fact that we wanted enough trials to run a neural network on it, we could not separate the different images categories (i.e., face vs non-face). This limits the interpretability of the results. In one of the next steps, it would be interesting to compare conditions containing only face affective pictures with conditions containing only non-face affective pictures separately.

The analysis of EEG data for backward priming effects is even more challenging because so far no empirically supported theory for precognition exists. We found two EEG studies in the precognition literature that, like our study, included supervised machine learning methods for analysis, i.e., techniques related to neural networks [49,50]. Bilucaglia et al. [49] used machine learning algorithms to classify EEG activity based on brain anticipatory activity. In two conditions participants had to either actively predict (with a behavioural response) the identity of upcoming visual and auditory stimuli (four possibilities) or only to passively predict them (without a behavioural response). In both conditions, the authors found stimulus-specific patterns of brain activity in a time window of 1300 ms before onset of the relevant stimulus. In this time window participants saw nothing except a cue, stating a stimulus was coming, and a blank interstimulus interval. They conducted a temporal (window) analysis and corrected their analysis for multiple testing. Mossbridge [50] followed a similar approach. Her participants had to respond to four visual and auditory stimuli with two different keys. Again stimulus-specific patterns of brain activity around 550 ms prior to the presentation of the stimulus were found. Due to the exploratory nature of her analysis, no correcting for multiple testing was done. The results of both studies suggest that EEG activity contains specific anticipatory, precognitive information that can be used to differentiate trials. However, given the very different experimental paradigms and dependent EEG variables, the findings restrict comparability with and hypothesis generation for the present study.

Another big challenge for the analysis of backward priming data in general and EEG backward priming data in particular, is a reoccurring and vividly discussed pattern in precognition research and in research about anomalous or parapsychological (psi) effects: Positive study findings can often not be reliably replicated [14,37,51–54], something that is not unlike the replication crises in classic experimental psychology [55]. This makes the formulation of specific hypotheses and the related definitions of ROIs additionally difficult. One reasonable explanation for this replication problem is that psi effects do simply not exist [56]. However, a huge number of case reports [15–18] as well as meta-analyses [9,10,57] supplement positive experimental studies, like the seminal Bem study [8] and the successful replications of Bem’s results [11,12], justify the consideration of an alternative approach to explain this replication problem: Let us postulate that psi effects can occur but can predominantly be produced by particularly gifted individuals, while the majority of participants are less gifted [58,59]. Similar to other faculties of the mind, such as musicality, one could imagine a continuum of capacity between gifted and ungifted individuals. Results would then depend on how many gifted individuals one would include by chance. Furthermore, gifted individuals may be rare in the population and being gifted may be a state rather than a trait variable. In this case, there would be no guarantee that the effect can be measured at the particular time point of the experiment in the lab.

### Forward priming ERP results

We found significant ERP differences at right parietal electrodes. Due to the fact that our significant differences occur quite late, we can exclude that these differences between congruent and incongruent conditions are low level visual effects. Instead, they seem to represent more complex processes. Our significant differences start at around 600 ms after target onset and end around 900 ms after target onset, suggesting our effects could be a Late Positive Component [5,6]. To the best of our knowledge, studies have not reported a right lateralization of the Late Positive Component like we have found. This difference in results could be due to the fact that the paradigms are slightly different. We have a word as a prime and a picture as a target, while others have words as both primes and targets or switch the prime for a picture and the word as the target [5–7,47].

The N400 ERP component is typically found in the context of processing word semantics [60]. Some priming studies using words for both the prime and the target, reported a priming-related modulation of the N400 amplitude [5,7,47]. We did not find an N400 effect in our results. This could be due to the fact that in our experiment, only the prime was a word, whereas the target was a picture. There are, however, other studies that also do not have evidence for an N400 priming effect in their results [6], indicating that this ERP component might not be as robust as the Late Positive Potential.

### Backward priming ERP results

Our exploratory EEG data analysis approach applying running t-tests revealed two small time windows of significance at the left central CP5 electrode. Although the effects are small, short in time and not corrected for multiple testing, the following is worth mentioning: The CP5 electrode is roughly located over the left supramarginal gyrus [61], which is being discussed as relevant during the processing of word phonology and semantics [62–64]. This is particularly remarkable, because our prime stimuli are words and their semantics are either related (congruent trials) or unrelated (incongruent trials) to our target images. If word semantics is indeed relevant, we should also expect a N400 ERP effect, which we did not find. This possible contradiction needs to be resolved in follow-up studies.

Obviously, these exploratory and uncorrected EEG results have to be regarded with caution and cannot be used to draw strong conclusions. However, they will serve us – and potentially also other groups – for hypothesis generation for follow-up EEG studies on Bem-like backward priming tasks.

### EEG data analysis with neural networks and its potential limitations

Our second exploratory EEG data analysis approach, based on neural networks, did not reveal further insight into potentially interesting neural processes related to forward and/or backward priming. As can be seen along the two axes in the graph of Fig 9, representing forward and backward priming neural network classification accuracy of EEG data, individual accuracy data points are equally distributed among the 50% accuracy value and show comparable variability.

We were particularly interested to check our accuracy outcomes for potential backward priming outliers from the sample distribution, potentially representing participants gifted in precognition. The maximally achieved accuracy value for backward priming accuracy is around 65%. The participant who achieved this value does not look like such a strong outlier, given the accuracy value distribution of our sample. In addition to this, this participant did not show stronger reaction time effects in the backward priming condition. Of course, applying neural networks is a heuristic approach. We have a lot of degrees of freedom in choosing one of many possible network architectures. It is also possible that another choice of network architecture and parameters might have resulted in better accuracies. Furthermore, although classification neural networks can provide very impressive performance results [65], the final performance of any classification neural network strongly depends on the relation between signal and noise in the data. Let us assume we have only a weak signal, that is restricted to a small time window and is only present in the data from one or two electrodes. If we include all electrodes and a broad time window of interest as input to the network, the signal will most probably not be detected. We found small grand mean amplitude differences between congruent and incongruent conditions in forward priming, and even smaller grand mean amplitude differences in the backward priming task. For the neural network analysis we trained networks on the level of individual participants with single EEG trials. Given the relatively weak effects in the grand mean, it is reasonable to assume that the lack of neural network results could be due to also weak signals or small signal-to-noise ratios in the single EEG trials. Additionally, we were limited by the computation power and time. Perhaps, training the networks longer would have resulted in the networks learning the differences between congruent and incongruent trials better.

In summary, despite this innovative new analysis approach not being very informative for us, we are convinced that these types of methods stay interesting for EEG analyses in general. We are also convinced that analyses on the level of individual participants which they nicely offer is an important alternative to the classical group statistics.

## Conclusions

In the present study we replicated the behavioural effects from the literature in an affective forward priming paradigm. We failed to replicate related behavioural backward priming effects as reported by Bem and others. We thus continued the replication problem which has been known for a long time in experimental psi research but which has also recently been shown to be widespread in other research areas [66,67]. Our EEG data point in the same direction as the behavioural data. We found EEG correlates of forward priming, which are a good confirmation of the few existing EEG studies on this topic. We also found some indication of effects in the ERP backward priming task. However, these effects were shorter in time, smaller than the forward priming effects, and would not survive correcting for multiple testing. Every now and then the psi literature reports about few outstanding participants with remarkable performances [68–70]. Such results point to trait effects in those individuals rather than state effects. Although, this questions the application of group statistics, it may be interesting to apply our approach of statistics within individual participants with a potentially refined version of our neural network approach.

With our specific experimental forward priming tasks, we complement earlier behavioural findings and are able to show a novel brain activity signature that is specific to the word-prime and photo-target setup (the lateralized Late Positive Component). With the backward priming task, we can only tentatively link specific brain activation to an area surrounding the left supramarginal gyrus. Only a follow-up EEG source analysis could verify to which extent the supramarginal gyrus is actually implicated. Such neural processing which could be seen as related to phonological and semantic word processing of the prime stimulus following the response would be meaningful given the specific task. However, all these results must be replicated with a hypothesis-driven analysis and only after confirmation in a follow-up study, which we are currently in the process of conducting, can one make firmer conclusions.

## Supporting information

S1 Table Number of incorrectly classified trials. (DOCX)

S2 Table Correlations between questionnaire items and behavioural and EEG variables. (DOCX)

S1 Fig CP5 difference ERP trace also showing Cohen’s d. (TIF)

S2 Fig P8 difference ERP trace also showing Cohen’s d. (TIF)

S3 Fig Freiburg Mindfulness Inventory (FMI) correlations with reaction time difference and ERP amplitude. Freiburg Mindfulness Inventory (FMI) correlations with reaction time difference (left column) and ERP amplitude average of the first time window (TW) of statistical significance at CP5 (see Fig. 5a). Both for the backward priming experiment. The x-axes depict the reaction time difference or the first time window (TW) amplitude in µV. The y-axes depict the questionnaire scores, showing either the scores of the “accept” questions, the “presence” questions or the sum of the two. Each participant is depicted in a different colour. In (a) one can see the Pearson correlations and in (b) the Spearman correlations. In (a) there is a significant negative correlation between reaction time difference and the presence questions. This significance is gone when calculating the correlations with the Spearman test (b). In (a) there is also a significant negative correlation between the ERP amplitude in the first time window of significance and the sum of the presence and accept questions. Again this significance is gone when using the Spearman test (b).
(TIF)
