# Supplementary material for: Behavioural and EEG correlates of forward and backward priming—An exploratory study
Source: PLoS One. 2025 May 8;20(5):e0322930. doi: 10.1371/journal.pone.0322930 (PMC12061123; doi:10.1371/journal.pone.0322930)
Supplement: S1 Table — (DOCX) [file pone.0322930.s001.docx]

| Participant Name | Forward Priming | | Backward Priming | |
| --- | --- | --- | --- | --- |
|  | Congruent | Incongruent | Congruent | Incongruent |
| P1 | 11 | 21 | 20 | 21 |
| P2 | 4 | 8 | 5 | 5 |
| P3 | 11 | 10 | 19 | 15 |
| P4 | 31 | 30 | 27 | 38 |
| P5 | 14 | 17 | 18 | 19 |
| P6 | 18 | 13 | 17 | 11 |
| P7 | 28 | 32 | 68 | 84 |
| P8 | 67 | 58 | 73 | 44 |
| P9 | 34 | 41 | 59 | 81 |
| P10 | 47 | 85 | 27 | 23 |
| P11 | 12 | 12 | 10 | 20 |
| P12 | 4 | 3 | 6 | 5 |
| P13 | 5 | 4 | 9 | 11 |
| P14 | 7 | 15 | 7 | 10 |
| P15 | 8 | 32 | 6 | 21 |
| P16 | 7 | 37 | 11 | 7 |
| P17 | 2 | 1 | 1 | 7 |
| P18 | 17 | 10 | 11 | 10 |
| P19 | 4 | 6 | 3 | 5 |
| P20 | 4 | 13 | 2 | 4 |
| P21 | 13 | 13 | 21 | 14 |
| P22 | 15 | 7 | 9 | 13 |
| P23 | 5 | 4 | 7 | 10 |
| P24 | 14 | 23 | 20 | 24 |
| P25 | 5 | 83 | 11 | 9 |
| P26 | 10 | 15 | 15 | 12 |
| P27 | 11 | 13 | 49 | 42 |
| P28 | 8 | 9 | 10 | 8 |
| P29 | 5 | 5 | 5 | 8 |
| P30 | 10 | 9 | 13 | 14 |
| P31 | 7 | 8 | 14 | 12 |

Number of incorrect trials for each participants, separated into forward priming and backward priming and congruent and incongruent.
