# Supplementary material for: Behavioural and EEG correlates of forward and backward priming—An exploratory study
Source: PLoS One. 2025 May 8;20(5):e0322930. doi: 10.1371/journal.pone.0322930 (PMC12061123; doi:10.1371/journal.pone.0322930)
Supplement: S2 Table — (DOCX) [file pone.0322930.s002.docx]

Forward Priming Variables – Spearman Correlation

| Questionnaire variable | Reaction Time  (rho, p-value) | Small TW  (rho, p-value) | Large TW  (rho, p-value) |
| --- | --- | --- | --- |
| External Phenomena | 0.0062, 0.97 | -0.23, 0.21 | -0.21, 0.26 |
| Internal Phenomena | -0.27, 0.14 | -0.21, 0.25 | -0.17, 0.36 |
| Phenomena of Coincidence | -0.068, 0.72 | -0.076, 0.68 | 0.029, 0.88 |
| Phenomena of Dissociation | 0.082, 0.66 | -0.036, 0.85 | 0.045, 0.81 |
| Accept | 0.059, 0.75 | 0.053, 0.78 | -0.075, 0.69 |
| Presence | 0.055, 0.77 | -0.043, 0.82 | -0.034, 0.86 |
| Sum | 0.19, 0.3 | 0.12, 0.56 | -0.044, 0.81 |
| Non-planning | -0.13, 0.47 | -0.066, 0.73 | 0.074, 0.69 |
| Motor | -0.17, 0.35 | -0.08, 0.67 | 0.01, 0.59 |
| Cognitive | 0.2, 0.52 | -0.13, 0.49 | 0.049, 0.79 |
| Total | -0.14, 0.47 | -0.17, 0.36 | 0.014, 0.94 |

Forward Priming Variables – Pearson Correlation

| Questionnaire Variable | Reaction Time  (r, p-value) | Small TW  (r, p-value) | Large TW  (r, p-value) |
| --- | --- | --- | --- |
| External Phenomena | -0.024, 0.9 | -0.36, 0.046 | -0.28, 0.13 |
| Internal Phenomena | -0.14, 0.46 | -0.28, 0.13 | -0.3, 0.1 |
| Phenomena of Coincidence | -0.13, 0.48 | -0.077, 0.68 | 0.043, 0.82 |
| Phenomena of Dissociation | 0.062, 0.74 | -0.096, 0.61 | 0.027, 0.89 |
| Accept | 0.13, 0.47 | -0.074, 0.69 | -0.16, 0.39 |
| Presence | 0.13, 0.49 | -0.072, 0.7 | 0.055, 0.77 |
| Sum | 0.27, 0.14 | 0.0018, 0.99 | -0.081, 0.67 |
| Non-planning | -0.074, 0.69 | -0.062, 0.74 | 0.011, 0.95 |
| Motor | -0.077, 0.68 | -0.11, 0.54 | -0.015, 0.93 |
| Cognitive | 0.15, 0.44 | -0.15, 0.44 | 0.024, 0.9 |
| Total | -0.022, 0.91 | -0.13, 0.48 | 0.0067, 0.97 |

Tables showing the Spearman (top table) and Pearson (bottom table) correlation values of the three questionnaires and the different forward priming variables (Reaction Time, EEG Amplitude Small Time Window and Large Time Window). The light gray rows correspond to the PExE questionnaire items, the medium gray with the BIS-11 items and then dark gray with the FMI items.

Backward Priming Variables – Spearman Correlation

| Questionnaire Variable | Reaction Time  (rho, p-value) | First TW  (rho, p-value) | Second TW  (rho, p-value) |
| --- | --- | --- | --- |
| External Phenomena | -0.18, 0.33 | -0.018, 0.92 | -0.14, 0.46 |
| Internal Phenomena | -0.33, 0.073 | -0.27, 0.15 | 0.012, 0.95 |
| Phenomena of Coincidence | 0.1, 0.58 | -0.12, 0.51 | 0.032, 0.86 |
| Phenomena of Dissociation | -0.23, 0.2 | -0.025, 0.89 | 0.27, 0.15 |
| Accept | 0.18, 0.34 | -0.18, 0.32 | -0.23, 0.22 |
| Presence | -0.31, 0.089 | -0.3, 0.096 | -0.14, 0.47 |
| Sum | -0.041, 0.83 | -0.26, 0.15 | -0.17, 0.35 |
| Non-planning | -0.057, 0.76 | 0.23, 0.21 | 0.33, 0.066 |
| Motor | -0.11, 0.54 | 0.062, 0.74 | 0.13, 0.5 |
| Cognitive | 0.01, 0.96 | 0.12, 0.53 | 0.29, 0.11 |
| Total | -0.02, 0.91 | 0.1, 0.59 | 0.22, 0.24 |

Backward Priming Variables – Pearson Correlation

| Questionnaire Variable | Reaction Time  (r, p-value) | First TW  (r, p-value) | Second TW  (r, p-value) |
| --- | --- | --- | --- |
| External Phenomena | -0.12, 0.52 | -0.012, 0.95 | -0.15, 0.42 |
| Internal Phenomena | -0.24, 0.19 | -0.26, 0.16 | -0.054, 0.77 |
| Phenomena of Coincidence | 0.053, 0.78 | -0.062, 0.74 | -0.011, 0.95 |
| Phenomena of Dissociation | -0.22, 0.24 | -0.076, 0.69 | 0.051, 0.79 |
| Accept | 0.13, 0.48 | -0.31, 0.086 | -0.31, 0.092 |
| Presence | **-0.42, 0.019** | -0.075, 0.69 | -0.02, 0.92 |
| Sum | -0.066, 0.72 | **-0.37, 0.041** | -0.25, 0.17 |
| Non-planning | -0.074, 0.69 | 0.23, 0.22 | 0.31, 0.094 |
| Motor | -0.12, 0.56 | -0.041, 0.83 | 0.24, 0.9 |
| Cognitive | 0.057, 0.76 | 0.073, 0.7 | 0.23, 0.21 |
| Total | 0.066, 0.72 | 0.12, 0.53 | 0.24, 0.19 |

Tables showing the Spearman (top table) and Pearson (bottom table) correlation values of the three questionnaires and the different backward priming variables (Reaction Time, EEG amplitude First Time Window and Second Time Window). The light gray rows correspond to the PExE questionnaire items, the medium gray with the BIS-11 items and then dark gray with the FMI items.
