# Supplementary figures and images for: Behavioural and EEG correlates of forward and backward priming—An exploratory study
Source: PLoS One. 2025 May 8;20(5):e0322930. doi: 10.1371/journal.pone.0322930 (PMC12061123; doi:10.1371/journal.pone.0322930)

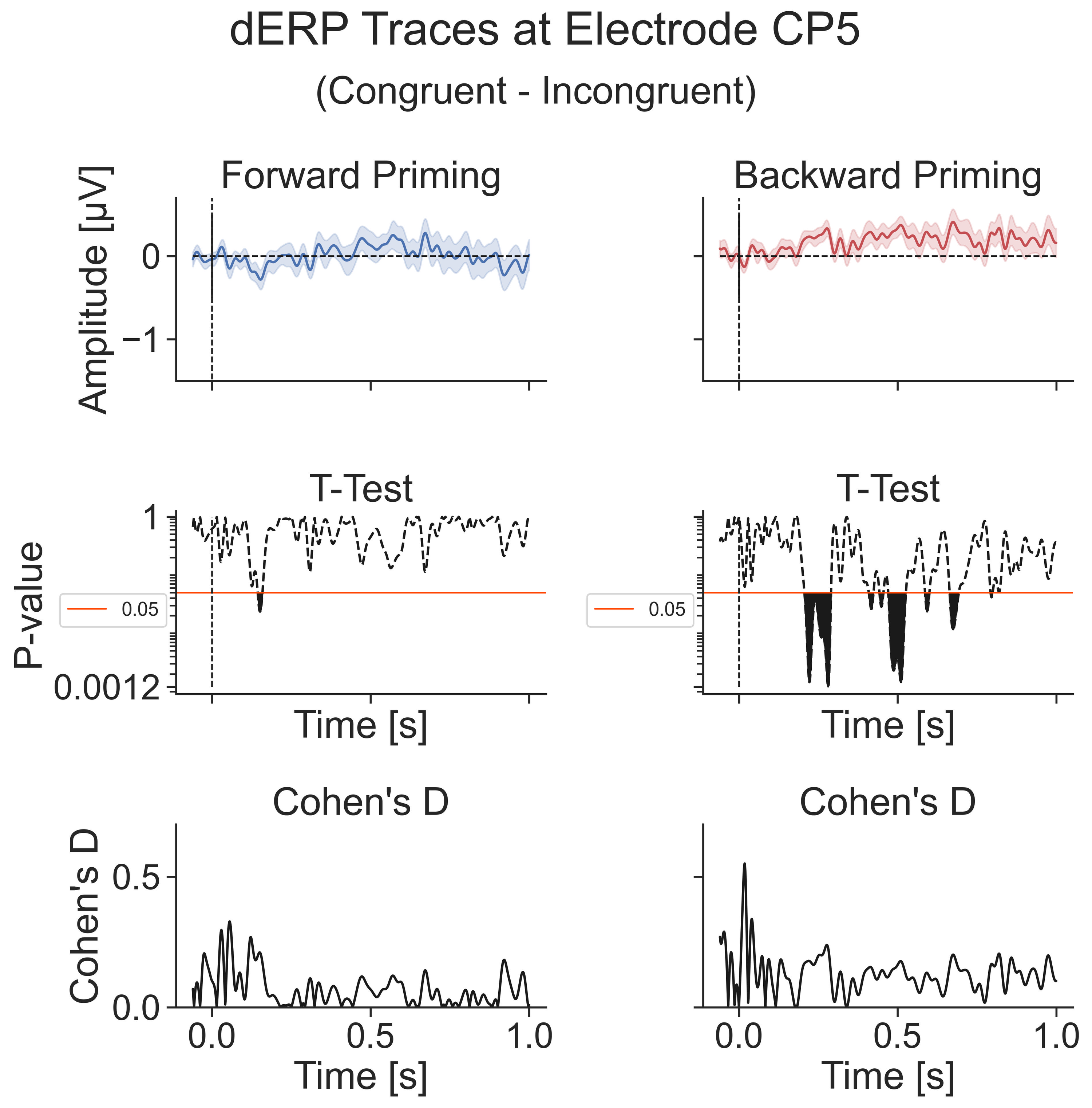

Supplement: S1 Fig — (TIF) [file pone.0322930.s003.tif]

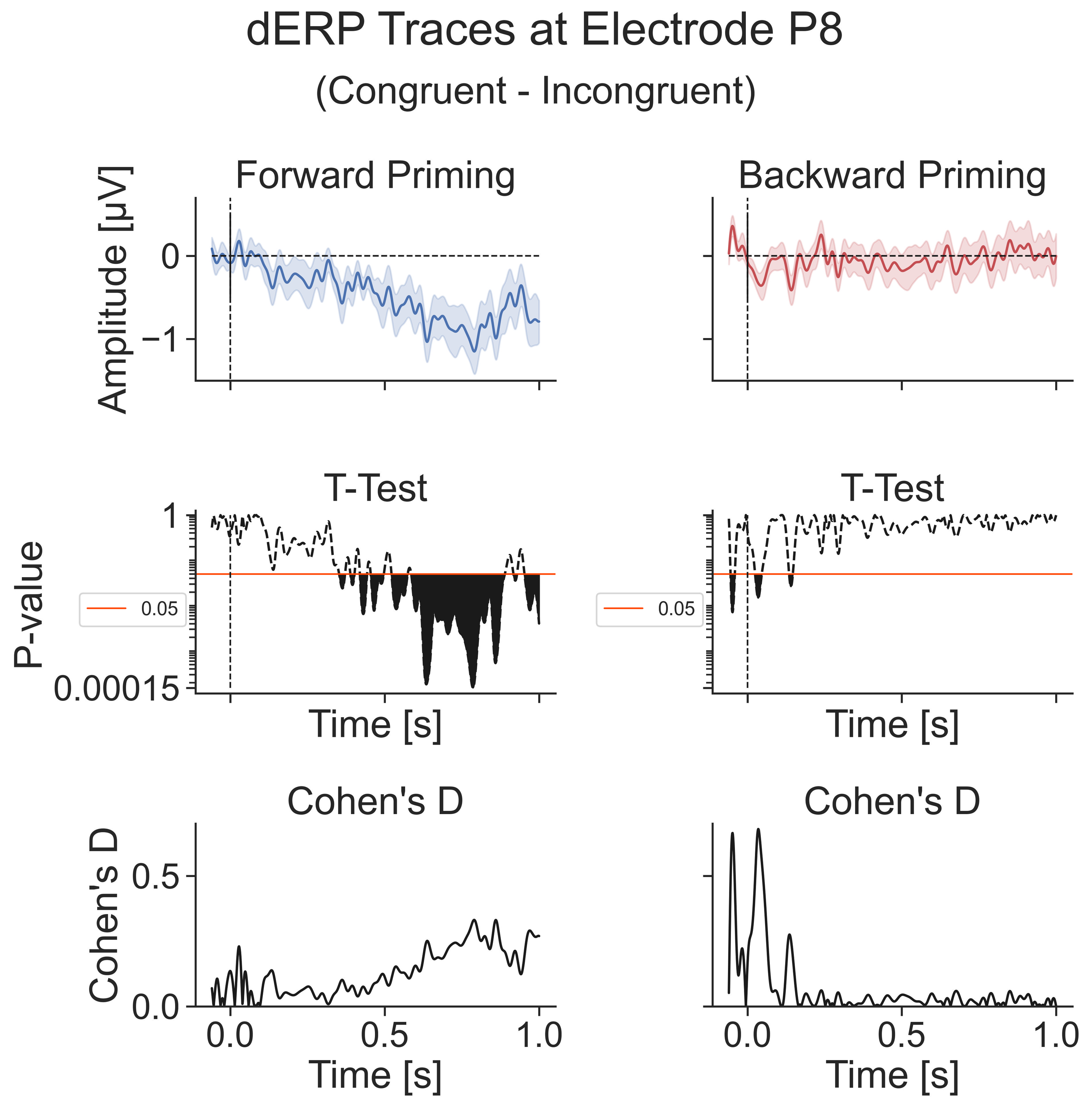

Supplement: S2 Fig — (TIF) [file pone.0322930.s004.tif]

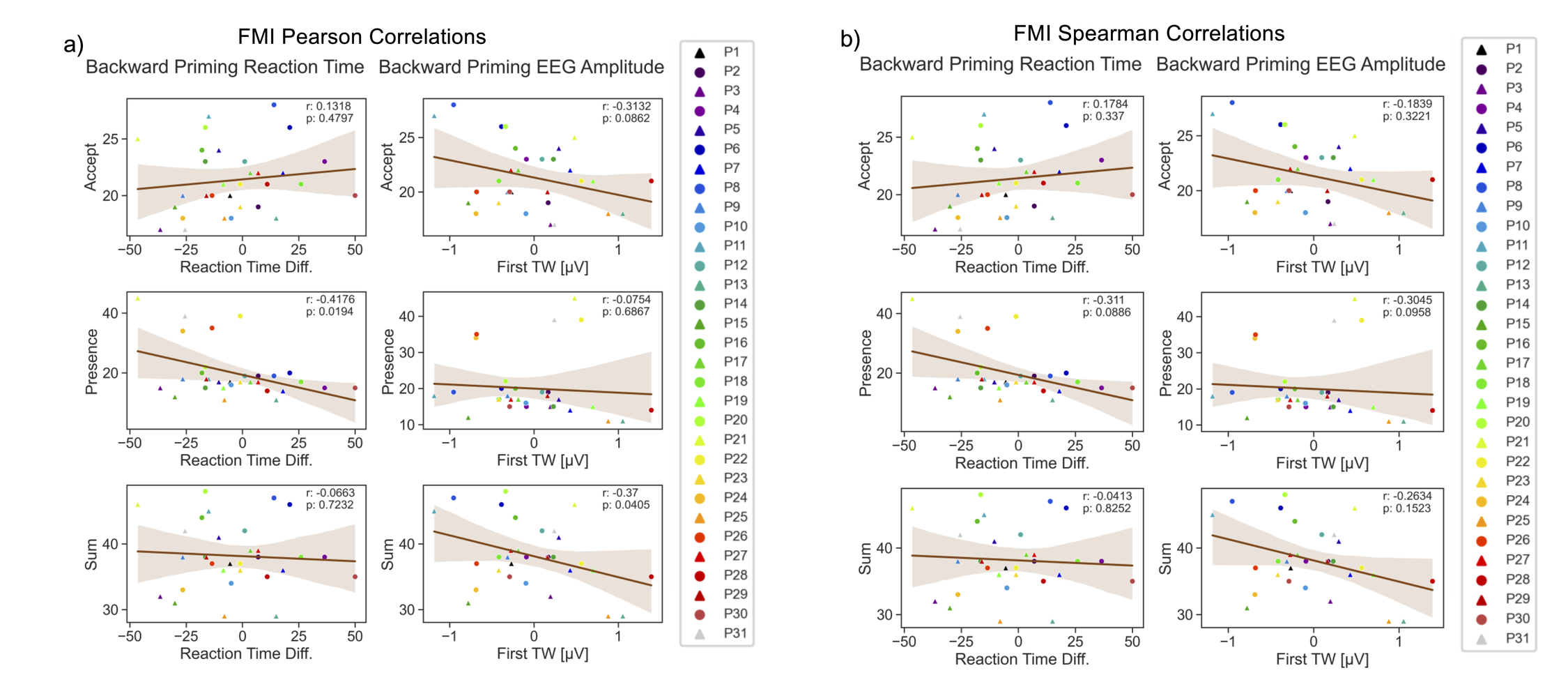

Supplement: S3 Fig — Freiburg Mindfulness Inventory (FMI) correlations with reaction time difference (left column) and ERP amplitude average of the first time window (TW) of statistical significance at CP5 (see Fig. 5a). Both for the backward priming experiment. The x-axes depict the reaction time difference or the first time window (TW) amplitude in µV. The y-axes depict the questionnaire scores, showing either the scores of the “accept” questions, the “presence” questions or the sum of the two. Each participant is depicted in a different colour. In (a) one can see the Pearson correlations and in (b) the Spearman correlations. In (a) there is a significant negative correlation between reaction time difference and the presence questions. This significance is gone when calculating the correlations with the Spearman test (b). In (a) there is also a significant negative correlation between the ERP amplitude in the first time window of significance and the sum of the presence and accept questions. Again this significance is gone when using the Spearman test (b). (TIF) [file pone.0322930.s005.tif]
